# Impacto del pretratamiento con inhibidores P2Y12 en pacientes con síndromes coronarios agudos sin elevación del ST. Análisis de dos registros multicéntricos

**DOI:** 10.47487/apcyccv.v4i3.322

**Published:** 2023-09-30

**Authors:** Alan R. Sigal, Mirza Rivero, Mayra Meza, Gerardo Filippa, Gastón Procopio, Camila M. Abud, Sebastián Nani, Martín Odone, Ernesto Duronto, Juan P. Costabel

**Affiliations:** 1 Instituto Cardiovascular de Buenos Aires. Buenos Aires, Argentina. Instituto Cardiovascular de Buenos Aires Buenos Aires Argentina; 2 CEMIC. Buenos Aires, Argentina. CEMIC Buenos Aires Argentina; 3 CEMEP. Tierra del Fuego, Argentina. CEMEP Tierra del Fuego Argentina; 4 Hospital Universitario Fundación Favaloro. Buenos Aires, Argentina. Hospital Universitario Fundación Favaloro Buenos Aires Argentina; 5 Clínica Olivos. Buenos Aires, Argentina. Clínica Olivos Buenos Aires Argentina; 6 Sanatorio San Lucas. Buenos Aires, Argentina. Sanatorio San Lucas Buenos Aires Argentina

**Keywords:** IMSEST, Tratamiento, Clopidogrel, NSTEMI, Treatment, Clopidogrel

## Abstract

**Objetivos.:**

Evaluar la tasa de uso de pretratamiento antiagregante en pacientes con síndrome coronario agudo sin elevación del segmento ST (SCASEST) y su asociación con eventos adversos en dos registros argentinos.

**Materiales y métodos.:**

Se analizaron retrospectivamente dos registros argentinos de síndromes coronarios agudos (SCA) de 2017 y 2022. Se exploró la incidencia de pretratamiento y el fármaco utilizado y se evaluó la relación entre esta estrategia y un resultado clínico compuesto de eventos intrahospitalarios: muerte + infarto de miocardio + trombosis del stent + angina postinfarto + accidente isquémico transitorio/accidente cerebro vascular más los eventos hemorrágicos (BARC 2 o superior). Posteriormente, se realizó un análisis multivariado por regresión logística con otras variables clínicas.

**Resultados.:**

Se incluyeron 1297 pacientes. El 75,6% eran hombres, 25,6% diabéticos, 27,1% fumadores, 70,3% hipertensos y 23,1% tenían un SCA previo. La edad media era de 55,3 años. La puntuación GRACE media fue de 113,5 y la CRUSADE de 23,8. El 44% de los pacientes recibieron pretratamiento, la mayoría con clopidogrel (93,5%). El pretratamiento se asoció significativamente con una mayor incidencia del resultado clínico compuesto (10,1% vs. 6,9%) (OR 1,56; IC 95%: 1,06-2,3; p=0,02). Los eventos hemorrágicos fueron numéricamente más frecuentes con el pretratamiento (8,7% frente a 5,9%) (OR 1,51; 0,99-2,3; p=0,054). En el análisis multivariado el pretratamiento ya no se asoció con una mayor incidencia de desenlaces isquémicos (OR 1,4; 0,89-2,3; p=0,13) ni hemorrágicos.

**Conclusiones.:**

El pretratamiento se utilizó en casi la mitad de los pacientes, principalmente con clopidogrel, y no mostró una reducción de eventos isquémicos en pacientes con SCASEST de la vida real.

## Abstract (en)

**Objectives.:**

To evaluate the rate of use of antiplatelet pretreatment in patients with non-ST elevated acute coronary syndrome (NSTEACS) and its association with adverse events in two Argentine registries.

**Materials and methods.:**

We retrospectively analyzed two Argentine acute coronary syndrome (ACS) registries from 2017 and 2022. We explored the incidence of pretreatment and the drug used. We evaluated the relationship between this strategy and a composite clinical outcome of in-hospital events: death + myocardial infarction + stent thrombosis + post-MI angina + transient ischemic event/cerebrovascular event, and with bleeding events (BARC 2 or higher). Subsequently, we performed a multivariate analysis by logistic regression with other clinical variables.

**Results.:**

A total of 1297 patients were included; 75.6% were men, 25.6% diabetics, 27.1% smokers, 70.3% hypertensive, and 23.1% had a previous ACS. The mean age was 55.3 years. The mean GRACE score was 113.5, and the CRUSADE was 23.8. 44% of the patients received pretreatment, the majority with clopidogrel (93.5%). Pretreatment was significantly associated with a higher incidence of the composite clinical outcome (10.1% vs. 6.9%) (OR 1,56; IC 95%: 1,06-2,3; p=0,02). Bleeding events were numerically more frequent with pretreatment (8.7% vs. 5.9%) (OR 1,51; IC95%: 0,99 -2,3; p=0,054). In the multivariate analysis, pretreatment was no longer associated with a higher incidence of ischemic outcomes (OR 1,4; IC95%: 0,89-2,3; p=0,13).

**Conclusion.:**

Pretreatment was used in almost half of the patients, mainly with clopidogrel, and did not show a reduction in ischemic events in patients with NSTACS.

## Introducción

El pretratamiento en el síndrome coronario agudo sin elevación del segmento ST (SCASEST) se refiere a la administración de una dosis de carga de un inhibidor P2Y12 (clopidogrel, ticagrelor o prasugrel), previo a conocer la anatomía coronaria. Esta estrategia está fundamentada en lograr un nivel mayor de antiagregación plaquetaria ^1^ previo a la realización del cateterismo cardíaco y colocación de *stent*, con el objetivo de reducir el riesgo de trombosis del *stent*.

Sin embargo, el pretratamiento no ha demostrado de forma sistemática una reducción de eventos isquémicos, y tanto el ticagrelor ^2^ como el prasugrel ^3^ tienen un inicio de acción más precoz que el clopidogrel ^4^, con lo cual no es necesaria una administración precoz para su uso eficaz. Además, trae consigo un probable aumento del riesgo hemorrágico, sobre todo demostrado con prasugrel ^5^ y en caso de que el paciente tenga enfermedad de múltiples vasos y requiera resolución quirúrgica requerirá un mayor tiempo de internación hasta poder operarse, o enfrentarse a una cirugía con riesgo elevado de sangrado ^6^^-^^9^.

No conocemos la incidencia de uso de pretratamiento en nuestro medio, ni las características de los pacientes a los que se le administró. Tampoco se conoce la incidencia de eventos isquémicos o hemorrágicos en relación a la estrategia de pretratamiento. Por lo que el objetivo primario del estudio fue evaluar la tasa de utilización de pretratamiento en la población argentina, y el tipo de fármaco utilizado para esta estrategia. Como objetivo secundario se exploró si el uso de pretratamiento se asoció con la incidencia de eventos isquémicos intrahospitalarios y mortalidad mediante un punto final combinado que incluyó: muerte, infarto de miocardio, trombosis del *stent*, angina posinfarto y accidente isquémico transitorio/accidente cerebro vascular (AIT/ACV). Además, se buscó asociación entre el uso de pretratamiento y la presencia de sangrado *Bleeding Academic Research Consortium* (BARC) 2 o mayor, durante la internación.

## Materiales y métodos

### Diseño y población de estudio

Se realizó un análisis retrospectivo de dos registros prospectivos multicéntricos de síndrome coronario agudo en la República Argentina: el Buenos Aires I ^10^ y el registro de síndromes coronarios agudos en centros de Argentina (RESCAR) ^(^^11^. Ambos registros incorporaron pacientes de centros de alto volumen y complejidad, principalmente del área metropolitana de Buenos Aires, y fueron diseñados y llevados a cabo por el Consejo de Emergencias y Cardiología Crítica de la Sociedad Argentina de Cardiología.

El registro Buenos Aires I fue prospectivo observacional que incorporó pacientes durante diciembre de 2017 a julio de 2018. Incluyó 1100 pacientes consecutivos con SCASEST de 21 centros.

RESCAR fue un registro prospectivo observacional multicéntrico que incluyó pacientes durante enero y agosto de 2022. Se consideraron 984 pacientes con síndrome coronario agudo con o sin elevación del segmento ST de 15 centros.

Para el presente análisis, se incluyeron pacientes de ambos registros que tuvieran diagnóstico de SCASEST, y se excluyeron aquellos con uso de tienopiridinas al ingreso por el evento índice y aquellos a los que no se les realizó tratamiento invasivo.

### Análisis estadístico

Para este análisis se utilizó el *software* IBM SPSS Statistics for Windows, version 25.0. Las variables continuas fueron expresadas como media y desvío estándar, o mediana y rango intercuartilo de acuerdo con las características de su distribución. Para el análisis de la normalidad se utilizó la prueba de Kolmogorov-Smirnov o Shapiro-Wilk, según correspondiera. Las variables categóricas se analizaron mediante la prueba de chi cuadrado o la prueba de Fisher, en tanto que las variables numéricas mediante la prueba de Student o test de U de Mann-Whitney, de acuerdo con su distribución.

Posterior al análisis univariado de ambos objetivos secundarios, se realizó un análisis mutivariado por regresión logística incluyendo todas aquellas variables que presentaron un valor de p < 0,10 en el análisis univariado. Los resultados se expresaran en odds ratio (OR) con intervalos de confianza de 95%. Se consideró con significancia estadística un error tipo I menor o igual que el 5% (p <0,05 a dos colas).

### Consideraciones éticas

Este estudio de datos secundarios se llevó a cabo en cumplimiento con la Ley Nacional de Protección de Datos Personales N.° 25326. El estudio fue conducido de acuerdo con las normas éticas nacionales (Ley CABA N.° 3301, Ley Nacional de Investigación Clínica en Seres Humanos, Declaración de Helsinki y otras).

## Resultados

**Figura 1:**
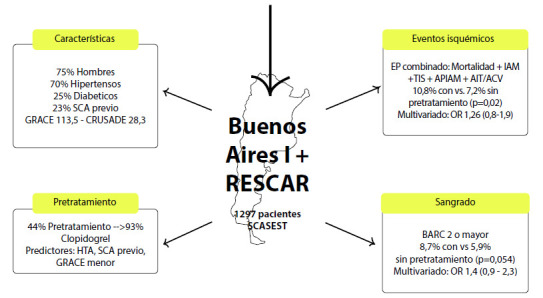
Figura central que resume los hallazgos del trabajo.

Se incluyeron 1297 pacientes con una mediana de edad de 55,3 años. El 75,6% eran hombres, el 25,6% diabéticos, el 27,1% fumadores, el 70,3% hipertensos y el 23,1% tenían un síndrome coronario agudo previo **(**Figura 1**).**

SCA= síndrome coronario agudo. HTA=hipertensión arterial. EP=endopoint primario. IAM=infarto de miocardio. TIS= Trombosis intra-stent. APIAM= angina post infarto. AIT= accidente isquémico transitorio. ACV= accidente cerebrovascular.

El 70% de los SCASEST fueron infartos y el 30% restante anginas inestables. La puntuación GRACE (Global Registry of Acute Coronary Events) mediana fue de 113,5 (94-136) y la CRUSADE (Can Rapid Risk Stratification of Unstable Angina Patients Suppress Adverse Outcomes with Early Implementation of the ACC/AHA Guidelines) fue de 23,8 (13-33). La tasa de eventos intrahospitalarios resultó de 8,3%.

El 44% de los pacientes recibieron pretratamiento, el 93,5% con clopidogrel y el 6,5% con ticagrelor. La tasa de uso de pretratamiento varió significativamente entre centros, oscilando entreel 10 al 60%. La mediana de tiempo a la cinecoronariografía fue de 18 (12-23) h. El 12% (155 pacientes) requirió cirugía coronaria dentro de la internación índice.

Los pacientes que recibieron pretratamiento eran más frecuentemente hipertensos (73,8% vs. 67%; p=0,01), menos dislipémicos (54,2% vs. 63,9%; p=0,001) y tenían una mayor incidencia síndrome coronario agudo (SCA) previo (27% vs. 19%; p=0,002). También eran más jóvenes (57 vs. 64 años de mediana; p=0,001), y en menos oportunidades recibían anticoagulación oral (4,7% vs. 7,4%; p=0,04). El resto de las características basales de los pacientes eran similares entre aquellos que recibieron pretratamiento y aquellos que no (Tabla 1).

**Tabla 1:** Características basales de la población según si recibieron o no pretratamiento

Variable	Sin pretratamiento n=724	Con pretratamiento n=573	Valor de p
Sexo masculino - n (%)	543 (75)	438 (76)	0,54
Edad - años, mediana (P25-75)	64 (47-75)	57 (35-72)	0,001
IMC - kg/m^2^, mediana (P25-75)	28,1 (25-33,1)	28,7 (26-35,2)	0,660
Hipertensión - n (%)	489 (67,5)	423 (73,8)	0,010
Diabetes - n (%)	173 (23,8)	159 (27,7)	0,110
Dislipemia - n (%)	463 (63,9)	311 (54,2)	0,001
Tabaquismo - n (%)	212 (29,3)	140 (24,4)	0,051
Fey - %, mediana (P25-75)	50 (20-60)	48 (18-55)	0,150
CRUSADE - valor, mediana (P25-75)	24 (13-33)	22 (13,7-32)	0,530
GRACE - valor, mediana (P25-75)	114 (94-136)	111,5 (90-131)	0,930
Sangrado previo - n (%)	8 (1,1)	7 (1,2)	0,840
SCA previo - n (%)	144 (19,8)	155 (27)	0,002
ATC alejada - n (%)	153 (21,1)	124 (21,6)	0,820
CRM previa - n (%)	65 (8.9)	53 (9,2)	0,860
FA previa - n (%)	60 (8,3)	39 (6,8)	0,320
ACV previo - n (%)	41 (5,6)	30 (5,2)	0,730
EVP previa - n (%)	45 (6,2)	35 (6,1)	0,930
Aspirina previa - n (%)	296 (40,1)	252 (43,9)	0,262
ACO prev - n (%)	54 (7,4)	27 (4,7)	0,040
KK			0,460
I	672 (92,8)	528 (92,1)	
II	36 (4,9)	37 (6,4)	
III	13 (1,8)	7 (1,2)	
IV	3 (0,5)	1 (0,1)	

IMC: índice de masa corporal. SCA: síndrome coronario agudo. ATC: angioplastia transluminal coronaria. CRM: cirugía de revascularización miocárdica. FA: fibrilación auricular. ACV: accidente cerebrovascular. EVP: enfermedad vascular periférica. ACO: anticoagulación oral. KK: Killip y Kimbal. FEy =Fracción de eyección

En cuanto a los objetivos secundarios, en el análisis univariado, el uso de pretratamiento se asoció con una mayor incidencia del punto final combinado de eventos intrahospitalarios, ocurriendo en 10,8% de los pacientes que recibieron pretratamiento, y en 7,2% de los que no lo recibieron (OR 1,56; IC 95%: 1,06-2,03; p=0,02). Respecto a los eventos hemorrágicos BARC 2 o mayores, se observó una tendencia a una mayor incidencia de estos, que no alcanzó significancia estadística, siendo de 8,7% en aquellos que recibieron pretratamiento y de 5,9% en los que no (OR 1,51; IC 95%: 0,99-2,3; p=0,054).

En análisis multivariado por regresión logística, el uso de pretratamiento no demostró asociación estadísticamente significativa con el punto final de eventos isquémicos intrahospitalarios (OR 1,26; IC 95%: 0,8-1,9) (Tabla 2). Respecto al punto de sangrado BARC 2 o mayor, el uso de pretratamiento tampoco se asoció estadísticamente con una mayor incidencia tras el análisis multivariado (OR 1,4; IC 95%: 0,9-2,3); (Tabla 3).

**Tabla 2:** Resultado de análisis multivariado del punto final combinado de eventos isquémicos intrahospitalarios

Variable	OR (IC 95%)	Valor de p
Uso de pretratamiento	1,26 (0,82-1,92)	0,277
Edad/año	0,99 (0,99-1,01)	0,905
Sexo masculino	1,03 (0,62-1,72)	0,889
Diabetes	1,31 (0,83-2,07)	0,243
Hipertensión arterial	1,00 (0,6-1,6)	0,980
Dislipemia	0,66 (0,42-1,01)	0,055
Sangrado previo	2,58 (0,61-10,76)	0,193
SCA previo	2,51 (1,55-4,07)	0,001
Aspirina previa	0,85 (0,53-1,36)	0,497
ACO previa	0,73 (0,36-1,48)	0,390
KK	2,63 (1,83-3,79)	0,001
FEVI	1,00 (0,99-1,01)	0,414

SCA: síndrome coronario agudo. ACO: anticoagulación oral. KK: Killip y Kimbal. FEVI: fracción de eyección del ventrículo izquierdo.

**Tabla 3:** Resultado de análisis multivariado del punto final combinado de eventos hemorrágicos intrahospitalarios

Variable	OR (IC95%)	p
Uso de pretratamiento	1,44 (0,89-2,33)	0,131
Edad/año	0,99 (0,98-1,00)	0,817
Sexo masculino	0,71 (0,41-1,21)	0,211
Diabetes	0,84 (0,47-1,47)	0,546
Hipertensión arterial	2,15 (1,13-4,1)	0,020
Dislipemia	0,83 (0,52-1,3)	0,470
Sangrado previo	4,16 (1,01-17,16)	0,048
SCA previo	0,98 (0,54-1,76)	0,954
Aspirina previa	1,57 (0,94-2,64)	0,084
ACO previa	0,92 (0,43-1,95)	0,830
KK	2,60 (1,76-3,83)	0,001
FEVI	0,99 (0,97-1,00)	0,105

SCA: síndrome coronario agudo. ACO: anticoagulación oral. KK: Killip y Kimbal. FEVI: fracción de eyección del ventrículo izquierdo.

## Discusión

En este estudio observacional retrospectivo, sobre bases recolectadas prospectivamente en dos registros multicéntricos nacionales de síndrome coronario agudo, el uso de pretratamiento con clopidogrel en pacientes con SCASEST no se tradujo en una mejoría de eventos isquémicos intrahospitalarios, ni en un aumento del sangrado BARC 2 o mayor, aunque sí presentó una tendencia en este último punto. Nos interesaría resaltar algunos aspectos de nuestros hallazgos.

Primero, la tasa de uso de la estrategia de pretratamiento resultó del 44%, lo que representa un valor elevado, teniendo en cuenta que las guías actuales de práctica clínica le otorgan una recomendación clase III al uso rutinario o IIb en circunstancias seleccionadas. Las razones para esto se pueden encontrar en el alto uso de clopidogrel como inhibidor P2Y12, teniendo en cuenta que la estrategia de tratamiento en sala es farmacológicamente más atractiva cuando se usan inhibidores más potentes como ticagrelor o prasugrel.

Segundo, el pretratamiento se indicó más en pacientes hipertensos y con síndrome coronario agudo previo. En relación al primer factor, nos cuesta encontrar una relación que justifique este hallazgo, en el sentido de que sea una variable tomada a consideración por los médicos a la hora de indicar el pretratamiento, y podría estar explicada por los sesgos propios de un estudio observacional no aleatorizado. Por el contrario, el SCA previo aumenta significativamente el riesgo de recurrencia isquémica de los pacientes y, por otro, lado dichos pacientes tienen más chance de requerir una angioplastia coronaria y por ello pudo haber sido una situación considerada por los médicos tratantes para indicarla. A su vez, la edad fue menor entre los pacientes que recibieron pretratamiento, lo que podría estar asociado al riesgo de hemorragias y la necesidad de cirugía coronaria.

Tercero, el análisis por centros mostró una gran variabilidad, yendo del 10 al 60%, lo que podría estar manifestando diferentes protocolos de actuación más que una decisión individualizada paciente a paciente. Esto podría estar relacionado con la tasa de cirugías de cada centro, la disponibilidad de estudio de coronariografía o el grado de convencimiento del beneficio o perjuicio de esta práctica. Es importante mencionar que la tasa de pacientes que requirió cirugía durante la internación resultó del 12%, elevada en comparación con otros registros como el registro de la ESC (Sociedad Europea de Cardiología) que encontró una tasa de 9,2% de cirugía para países de bajos y medianos ingresos ^12^, y siendo esta ocurrencia uno de los hechos temido al momento de indicar pretratamiento.

Cuarto, el pretratamiento no se asoció con una reducción de eventos isquémicos, lo que resulta similar a lo mostrado por la literatura internacional. Por ejemplo, una cohorte Sueca ^13^ de casi 65 000 pacientes no encontró un beneficio en supervivencia a los 30 días o 1 año, o de la incidencia de trombosis del *stent* con el uso de pretratamiento, aunque sí un aumento de eventos hemorrágicos manifestado por un aumento del 50% del riesgo relativo de sangrados intrahospitalarios. Los ensayos que favorecieron el uso de pretratamiento son en su mayoría antiguos, casi exclusivamente con clopidogrel, y reflejo de una práctica no utilizada actualmente en donde los tiempos de reperfusión en infarto agudo de miocardio sin elevación del segmento ST (IAMSEST) son cada vez menores. Por ejemplo, el estudio CREDO ^14^ encontró que el pretratamiento con clopidogrel obtuvo beneficio en los eventos a 28 días solamente cuando su uso precedió a la angioplastía por al menos 15 h. En un subanálisis del estudio CURE ^15^, el pretratamiento con clopidogrel demostró un beneficio en la reducción de eventos isquémicos a 30 días, pero la mediana de tiempo entre su uso y el cateterismo fue de 6 días. En nuestro trabajo el tiempo de pretratamiento fue relativamente corto, lo que podría haber impactado en la falta de beneficio de la estrategia. El estudio ARMYDA-5, que incluyó pacientes estables y un 39% de agudos, tampoco encontró beneficio con el uso de esta estrategia, ni tampoco en el análisis de subgrupo de los pacientes con síndrome coronario agudo ^16^. Una revisión sistemática de 2014 ^17^, incluyendo un total de 38 000 pacientes, encontró un beneficio en la reducción de MACE, pero principalmente dado por los estudios CREDO y CURE, sin beneficio en aquellos pacientes que se sometieron a angioplastía. Sin embargo, no encontró un beneficio en la reducción de mortalidad en el total de los pacientes, ni tampoco en la cohorte de pacientes que se sometieron a tratamiento invasivo. A su vez, encontró un aumento del riesgo de sangrado del 30-40% según los estudios analizados. Otro metaanálisis más reciente, que incluyó el DUBIUS y el ISAR-REACT, no encontró beneficio con el pretratamiento en el objetivo primario de MACE a 30 días, ni en los secundarios de muerte o infarto a 30 días, aunque sí encontró un aumento del sangrado mayor con un NND de 63 ^18^.

Es razonable que, en esta línea, las guías de práctica clínica de la AHA/ACC ^19^ no recomienden el uso de pretratamiento, mientras que las guías de la ESC de 2020 ^20^ lo recomienden como indicación IIB en aquellos pacientes con bajo riesgo de sangrado que no tienen planeado una estrategia invasiva precoz, contraindicando además el uso de prasugrel para esta estrategia, basado en los resultados del estudio ACCOAST en el cual el pretratamiento con prasugrel se tradujo en aumento de sangrado sin beneficio en eventos isquémicos. En la guía de síndromes coronarios agudos de la ESC de 2023 ^21^, esta recomendación cae a clase III en aquellos pacientes en los cuales se planea realizar una revascularización dentro de las 24 h, mientras que se mantiene como IIB para aquellos en los que se espera una demora mayor de 24 h y no tienen alto riesgo de sangrado, agregando la información del estudio DUBIUS ^22^ el cual fue finalizado prematuramente por futilidad de la estrategia de pretratamiento en pacientes que se realizarían un cateterismo dentro de las 72 h de ingreso por SCASEST.

En Argentina, el registro de síndromes coronarios agudos del Consejo Argentino de Residentes de Cardiología (CONAREC) del 2013 encontró que los pacientes que ingresaban con diagnóstico de IAMSEST recibían en un 85% de los casos clopidogrel al ingreso hospitalario ^23^. Un análisis del Buenos Aires I, previo al RESCAR, encontró que de los pacientes que recibieron inhibidores del receptor P2Y12, un 75% lo hicieron en forma de pretratamiento, pero esto no se tradujo en una reducción de eventos a 6 meses, ni a aumento de sangrado ^24^. Las revisiones de expertos coinciden en la ausencia de beneficio del uso sistemático de esta estrategia en base a la literatura internacional ^25^.

Las limitaciones de nuestro trabajo incluyen: el trabajo tiene los sesgos de la falta de randomización para justificar una relación directa entre el pretratamiento y los eventos. Es por ello que las conclusiones quedan en el campo de las hipótesis, mas allá del ajuste por ciertas variables conocidas.

En conclusión, los hallazgos van en línea con lo evidenciado en la literatura internacional actual, con una tasa de uso de pretratamiento de casi la mitad de los pacientes, sin poder hallar un beneficio con el uso sistemático de pretratamiento con clopidogrel en SCASEST.
